# Disruption of an amino acid transporter *LHT1* leads to growth inhibition and low yields in rice

**DOI:** 10.1186/s12870-019-1885-9

**Published:** 2019-06-20

**Authors:** Xiaohu Wang, Guangzhe Yang, Mingxing Shi, Dongli Hao, Qiuxing Wei, Zhigang Wang, Shan Fu, Yanhua Su, Jixing Xia

**Affiliations:** 1 State Key Laboratory of Conservation and Utilization of Subtropical Agro-bioresources, College of Life Science and Technology, Guangxi University, Nanning, 530005 China; 2 State Key Laboratory of Soil and Sustainable Agriculture, Institute of Soil Science, Chinese Academy of Sciences, No. 71, East Beijing Road, Nanjing, 210008 China

**Keywords:** Amino acid transporter, OsLHT1, Rice, Electrophysiology

## Abstract

**Background:**

Research on plant amino acid transporters was mainly performed in *Arabidopsis*, while our understanding of them is generally scant in rice. OsLHT1 (Lysine/Histidine transporter) has been previously reported as a histidine transporter in yeast, but its substrate profile and function *in planta* are unclear. The aims of this study are to analyze the substrate selectivity of OsLHT1 and influence of its disruption on rice growth and fecundity.

**Results:**

Substrate selectivity of OsLHT1 was analyzed in *Xenopus* oocytes using the two-electrode voltage clamp technique. The results showed that OsLHT1 could transport a broad spectrum of amino acids, including basic, neutral and acidic amino acids, and exhibited a preference for neutral and acidic amino acids. Two *oslht1* mutants were generated using CRISPR/Cas9 genome-editing technology, and the loss-of-function of OsLHT1 inhibited rice root and shoot growth, thereby markedly reducing grain yields. QRT-PCR analysis indicated that *OsLHT1* was expressed in various rice organs, including root, stem, flag leaf, flag leaf sheath and young panicle. Transient expression in rice protoplast suggested OsLHT1 was localized to the plasma membrane, which is consistent with its function as an amino acid transporter.

**Conclusions:**

Our results indicated that OsLHT1 is an amino acid transporter with wide substrate specificity and with preference for neutral and acidic amino acids, and disruption of OsLHT1 function markedly inhibited rice growth and fecundity.

**Electronic supplementary material:**

The online version of this article (10.1186/s12870-019-1885-9) contains supplementary material, which is available to authorized users.

## Background

Nitrogen (N) is one of the major mineral nutrients essential for plants growth and development. Plants absorb N mainly in the inorganic form (nitrate and ammonium) from soils, while the organic N such as amino acids could also be absorbed [1, 2]. Upon entering the plants, inorganic N is converted into amino acids in roots or leaves. The reduced N is transported mainly in the form of amino acids to developing vegetative or reproductive sink organs, where it is used for growth and development. The uptake and translocation of organic and inorganic N is mediated by a variety of transporters. Nowadays, the nitrate and ammonium transporters have been extensively studied in plants [3, 4], while the amino acid transporters are still poorly understood, especially in crop plants.

Physiological studies using isolated membrane vesicles and plant tissues have suggested the presence of multiple amino acid transporters with wide substrate specificity in plants [5–7]. Molecular methods have allowed the cloning of genes responsible for amino acids transport. The first plant amino acid transporters AAP1 and NAT2 were identified by functional complementation of yeast mutants defective in amino acid uptake from *Arabidopsis* cDNA libraries, both of which exhibited broad substrate specificity [8, 9]. Thereafter a multitude of amino acid transporters were reported from several plant species [10, 11]. In recent years, sequencing the full genomes of different plants gave us a chance to grasp the abundance of amino acid transporters in plant genomes. At least 67, 85 and 189 genes have been identified to encode putative amino acid transporters in *Arabidopsis*, rice and soybean, respectively [12–14]. Based on sequence analysis, the amino acid transporters identified so far could be divided into two families: the amino acid/polyamine/choline (APC) family and the amino acid transporter (ATF) family. The ATF family was also referred to as AAAP (amino acid/auxin permease) family, and encompasses at least five subfamilies: AAPs (amino acid permeases), LHTs (lysine/histidine transporters), ProTs (proline transporters), ANTs (aromatics and neutral amino acid transporters) and AUXs (auxin transporters) [13, 15, 16]. These transporters generally differ in substrate specificity/affinity and tissue localization [17].

AtLHT1, the first member identified in LHT family, was initially viewed as a lysine (Lys) and histidine (His) selective transporter [18]. However, subsequent studies with other LHT members revealed that LHTs could transport a broad but distinct spectrum of amino acids [19–21]. *LHT* genes were expressed in various plant organs, including roots, leaves and flowers. They could mediate not only amino acids uptake from soil, but also translocation and partitioning of amino acids within plants [19–21]. However, it should be noted that the research on LHT transporters was mainly performed in *Arabidopsis*, while our understanding of LHTs in rice is generally scant. A genome-wide survey identified 6 LHT members in rice [14]. Liu et al. [22] isolated a LHT family member *OsHT1* from rice by screening cDNA library, which could restore the growth of yeast mutant defective in His uptake. Due to highest identity with *AtLHT1* among the 10 LHT family members in *Arabidopsis*, *OsHT1* is referred to as *OsLHT1* hereafter.

Although OsLHT1-mediated His transport was determined in yeast [22], it is not clear at present whether OsLHT1 could transport other amino acids. In this study, the substrate specificity of OsLHT1 was analyzed in *Xenopus* oocytes, and we found that OsLHT1 was able to transport a broad spectrum of amino acids with preference for neutral and acidic amino acids. Furthermore, to explore the role of *OsLHT1* in rice growth and development, two *oslht1* mutants were constructed using the CRISPR-Cas9 gene-editing technology. It is found that disruption of *OsLHT1* significantly inhibited the rice growth and fecundity. Also, the expression pattern and subcellular localization of OsLHT1 was analyzed. This study will further our understanding of the substrate specificity of OsLHT1 and its roles in rice growth promotion and yield formation.

## Results

### Broad substrate specificity of OsLHT1

**Fig. 1:**
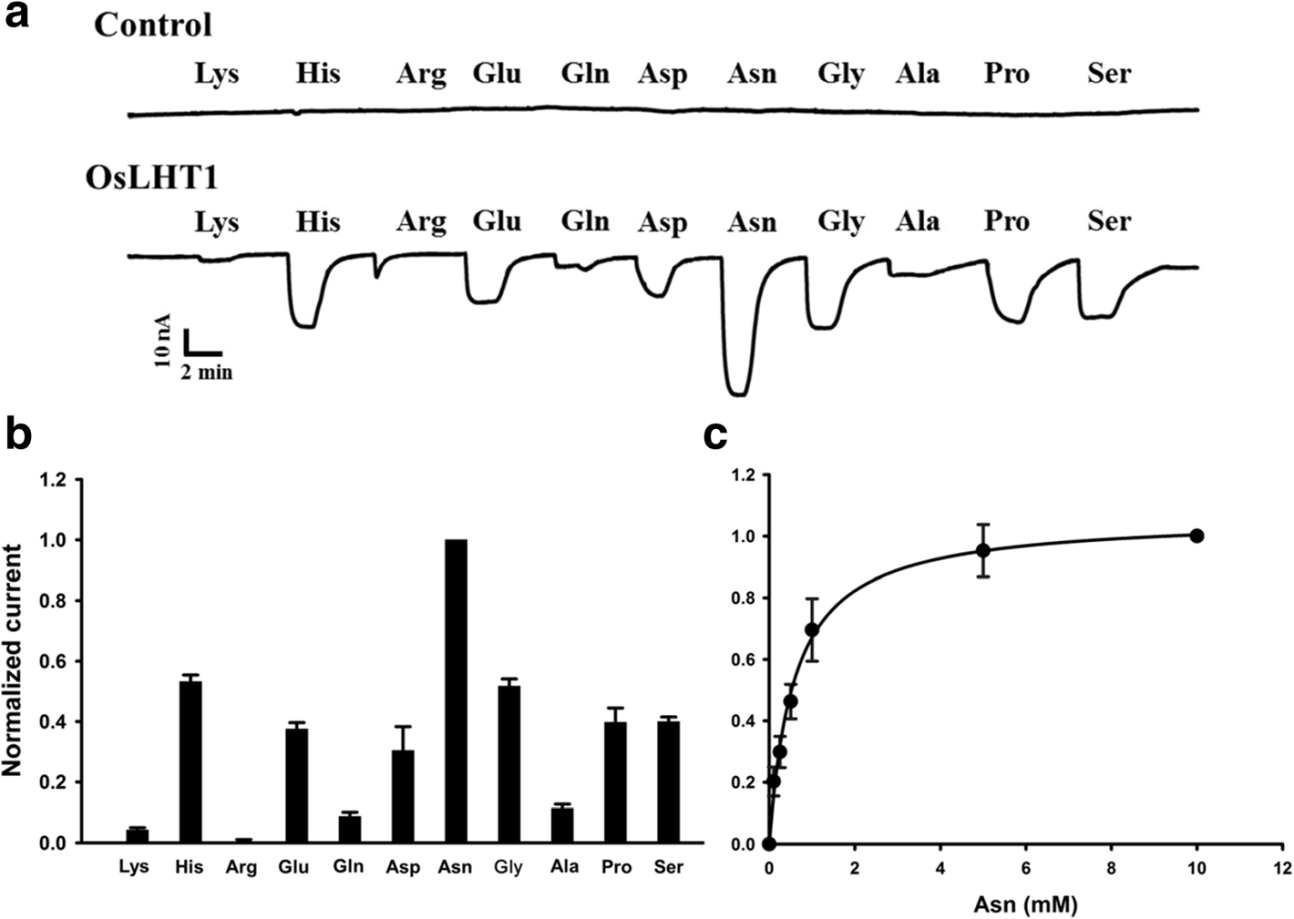
Substrate specificity of OsLHT1. **a** Representative inward currents induced by amino acids. Oocytes injected with water (upper trace, control) and OsLHT1 cRNA (lower trace) were voltage-clamped at − 70 mV and superfused with different amino acids (10 mM, pH 5.4). **b** Amino acid transport activity of OsLHT1. Oocytes expressing OsLHT1 were voltage-clamped at − 70 mV and superfused with different amino acids (10 mM, pH 5.4). Substrate-induced currents (background subtracted) were normalized to the Asn-induced current. Data are shown as means ± SD of six independent cells. **c** Kinetic analysis of Asn transport by OsLHT1. Asn-induced currents were recorded at − 140 mV (holding potential of − 40 mV) in different Asn concentrations. Net Asn-induced currents (background subtracted) were normalized to that obtained in 10 mM Asn, and then fitted to the Michaelis-Menten equation, as shown by the solid line. Data are shown as means ± SD of six independent cells

To analyze the substrate selectivity of OsLHT1, *OsLHT1* cRNA was injected into *Xenopus* oocytes, and two-electrode voltage clamp (TEVC) was employed to record the currents induced by different amino acids [23, 24]. Consistent with its function as a His transporter [22], OsLHT1 could transport His in oocytes (Fig. 1a). Besides His, several other amino acids such as Glu, Asp, Asn, Gly, Pro and Ser, could also be efficiently transported by OsLHT1, while Lys, Gln and Ala were only marginally transported (Fig. 1a, b). In contrast, water-injected control oocytes showed no current responses to external applications of amino acids (Fig. 1a). Unlike AtLHT1 [18], the neutral amino acid Asn was the best substrate for OsLHT1 among all of the amino acids tested (Fig. 1a, b). The kinetics of Asn transport by OsLHT1 was further analyzed in *Xenopus* oocytes, which is saturable with a *K*_m_ value of 583 ± 51 μM (Fig. 1c). These data demonstrate that OsLHT1 is an amino acid transporter with wide substrate specificity and with preferential selectivity for neutral and acidic amino acids.

### Knockout of *OsLHT1* results in growth inhibition

**Fig. 2:**
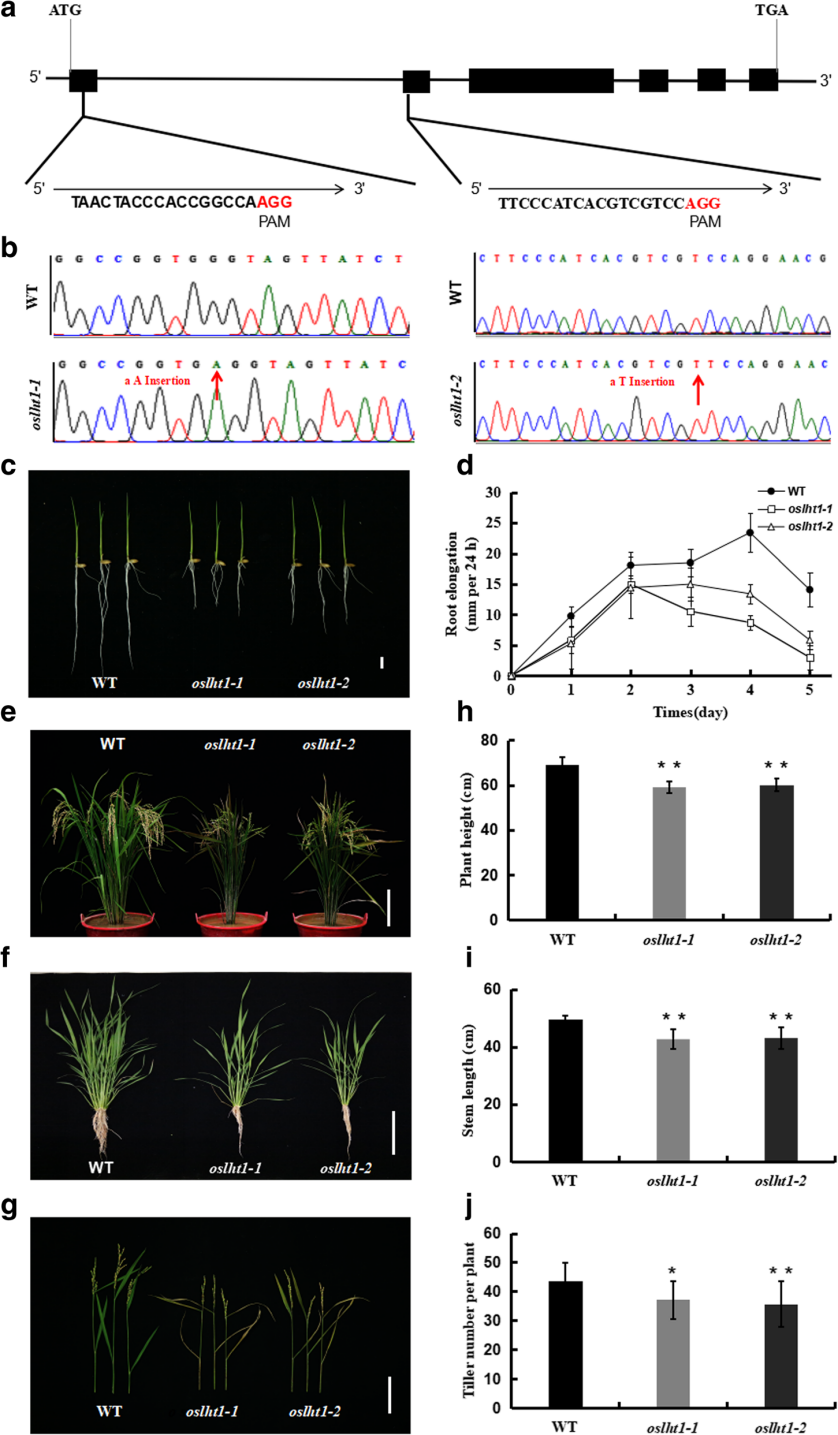
Targeted mutagenesis of OsLHT1 by CRISPR/Cas9 led to rice growth inhibition. **a** Gene structure of *OsLHT1* and the two targeted sites. Black boxes indicate exons. Red letters indicate the PAM of the recognition sequence. **b** Sequencing chromatography of wild type, mutants *oslht1–1* and *oslht1–2*. Red arrows indicate mutation sites. **c** Phenotype of 5-day-old WT, *oslht1–1* and *oslht1–2* grown on 0.5 mM CaCl_2_ solution. Bar =1 cm. **d** Time-dependent root elongation. Germinated seedlings were exposed to a 0.5 mM CaCl_2_ solution and the root length was measured at different days. Error bars represent ± SD (*n* = 10). **e**-**f** Gross morphological phenotypes of WT, *oslht1–1*and *oslht1–2* mutants grown in the field. Bar = 30 cm (e), 20 cm (f). **g** Symptoms of early senescence were present in *oslht1* plants at reproductive growth stage. Bar = 20 cm. **h**-**j** Comparison of plant height (h), stem length (i) and tiller number (j) of the wild type (WT) and *oslht1* plants at harvest. Values (h to j) are the mean ± SD (*n* = 15). Asterisks indicate significant differences from the wild type (*P < 0.05; **P < 0.01 by Student’s *t*-test)

To investigate the effect of *OsLHT1* disruption on rice growth, the CRISPR-Cas9 approach was employed to generate *oslht1* mutants. A CRISPR-Cas9 vector expressing guide RNAs targeting the coding region of *OsLHT1* was constructed (Fig. 2a) and transformed into rice (*Japonica* cv Nipponbare). Two mutants *oslht1–1* and *oslht1–2* were obtained, and then the genomic DNA of the two mutants was extracted for PCR amplification. Sequencing analysis of PCR products revealed that these two mutants were homozygous and had 1-bp adenine (A) and thymine (T) insertions at the desired target sites, respectively (Fig. 2b). Consequently, mutation of *oslht1–1* led to the conversion of Pro10 in OsLHT1 into Leu and subsequent frameshift; mutation of *oslht1–2* led to the conversion of Phe32 in OsLHT1 into Ser and subsequent frameshift. We also tested for potential off-target cleavage by searching the rice genome for sequences with high similarity to the *OsLHT1* target sequence using the on-line tool (http://skl.scau.edu.cn/offtarget/). For each target, four of the most likely off-target sites were examined, and no off-target cleavage was detected. Additionally, the transcript level of *OsLHT1* was detected, which was significantly reduced in the two *oslht1* lines compared with WT (Additional file 1: Figure S1).

When sowing seeds, the germination rates of *oslht1* mutants were lower than that of the wild type (WT) at 2 day after sowing (DAS), while the results are almost the same between the wild type and mutants at 3–5 DAS (Additional file 1: Figure S2). At 5 DAS, the shoot growth of the mutants is the same as the wild-type, but the mutants had much shorter roots than the wild type (Fig. 2c, d). These results suggested that the loss of OsLHT1 function slightly delayed seed germination, but markedly influenced root development. To evaluate the influence of OsLHT1 disruption on the growth over the entire growth period, the wild type and mutants were grown in soil. Compared with the wild type, both mutants *oslht1–1* and *oslht1–2* exhibited growth inhibition to different extents (Fig. 2e, f). Then the agronomic traits of wild-type and *oslht1* plants were further analyzed with respect to plant height, stem length and tiller number. The results showed that, compared with the wild type, the plant height, stem length and tiller number were reduced significantly in the *oslht1* plants (Fig. 2h, i, j). Additionally, during reproductive growth stage, premature senescence was also observed in the *oslht1* plants (Fig. 2g). These results indicated that a lack of OsLHT1 function affected plant normal growth and development.

### Knockout of *OsLHT1* results in low grain yields

**Fig. 3:**
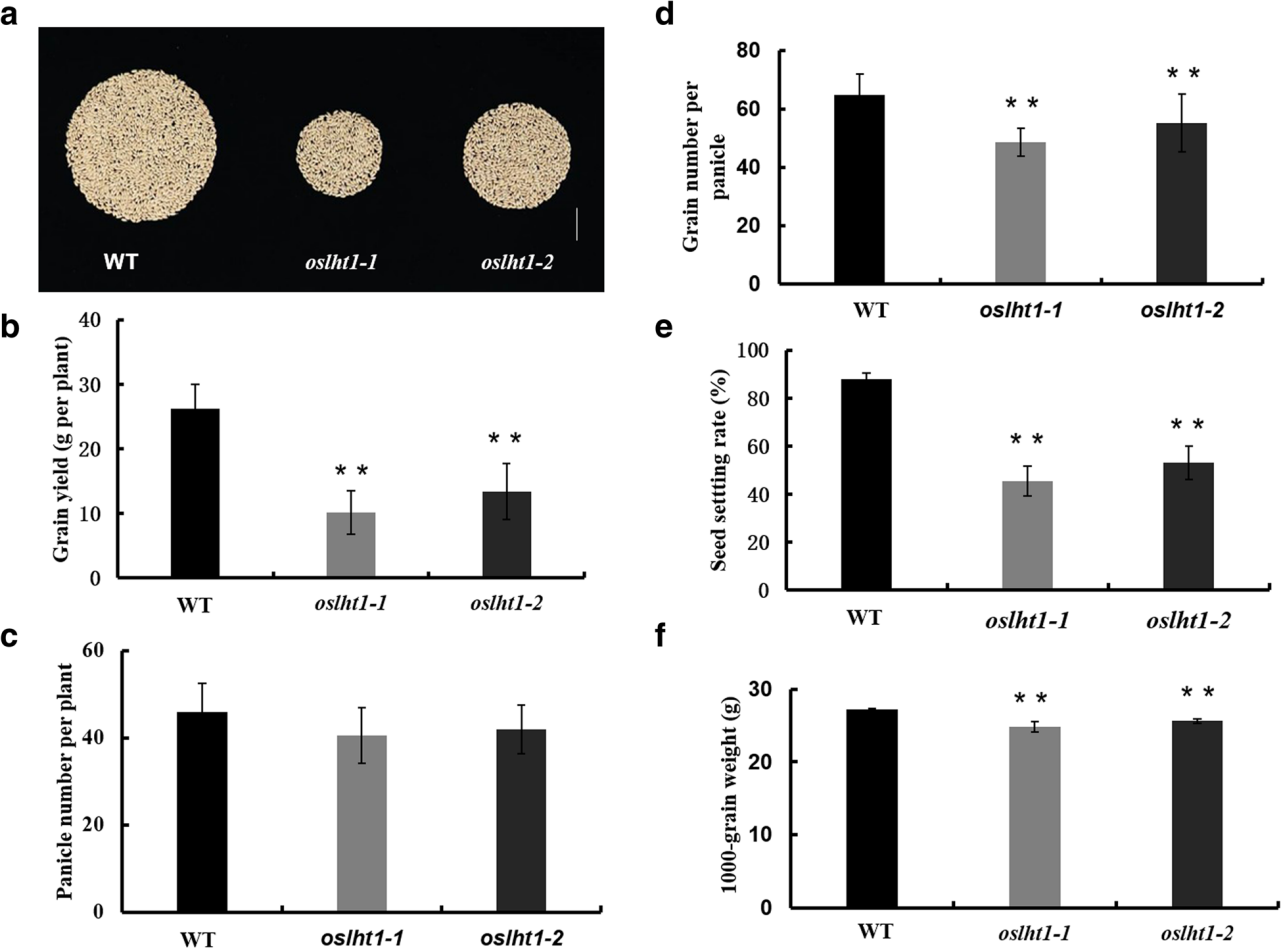
Loss of OsLHT1 function reduced rice grain yield. **a** Total grains per plant of WT and *oslht1* plants grown in field. Scale bars, 1 cm. **b**-**f** Comparison of grain yield per plant (**b**), panicle number per plant (**c**), grain number per panicle (**d**), seed setting rate (**e**) and 1000-grain weight (**f**) of the WT and *oslht1* plants. Values (b to f) are the mean ± SD (*n* = 15). Asterisks indicate significant differences from the wild type (*P < 0.05; **P < 0.01 by Student’s *t*-test)

To evaluate the influence of *OsLHT1* disruption on the grain yields, we compared the grain yields of *oslht1–1*, *oslht1–2* and the wild type. We found that the grain yields per plant of *oslht1* were decreased by 67.8 and 42.5% depending on the mutant line (Fig. 3a, b). Then the yield components, including panicle number per plant, grain number per panicle, seed setting rate and 1000-grain weight, were examined in detail. No significant differences in panicle number per plant were observed between *oslht1* and wild-type plants (Fig. 3c). The grain number per panicle of *oslht1–1* and *oslht1–2* compared with wild-type plants was decreased significantly, by 25 and 14%, respectively (Fig. 3d). The seed-setting rate was substantially reduced by 42.2% in *oslht1–1* and 34.7% in *oslht1–2* compared with WT (Fig. 3e), while the 1000-grain weight were reduced between 5.8 and 8.7% depending on the mutant (Fig. 3f). Additionally, no significant change was observed in grain size and shape in *oslht1* plants (Additional file 1: Figure S3). Taken together, these results suggested that disruption of *OsLHT1* had a large impact on grain number per panicle and seed setting rate, thus resulting in low yields in *oslht1* plants.

### Spatial expression and subcellular localization of OsLHT1

**Fig. 4:**
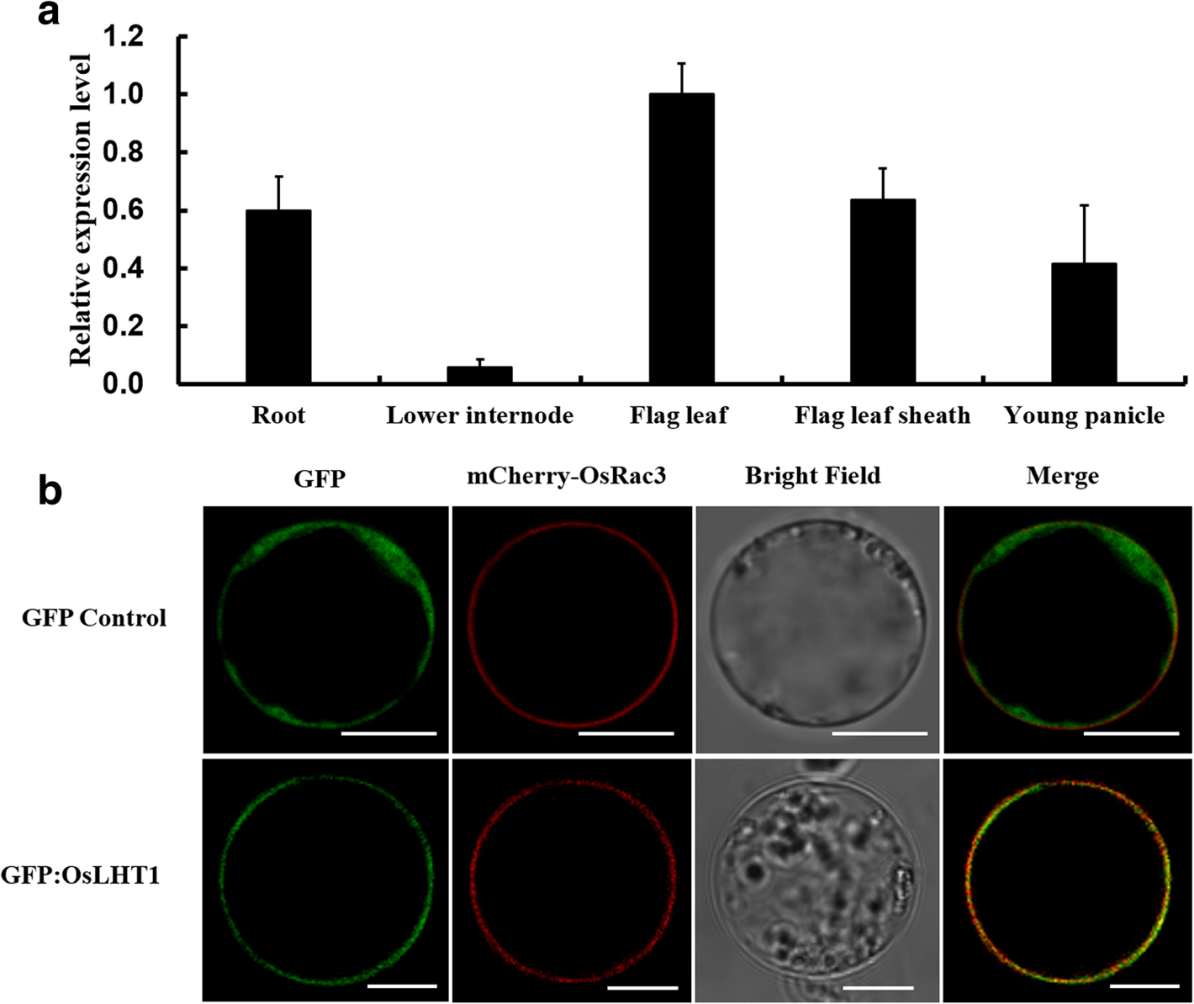
Organ-specific expression and subcellular localization of OsLHT1. **a** Expression of *OsLHT1* in various organs of the wild-type plants, analyzed by quantitative real-time PCR. The data are shown as the mean ± SD (*n* = 3). **b** Subcellular localization of the OsLHT1. *GFP:OsLHT1* or *GFP* was transiently introduced into rice protoplast together with *mCherry-OsRac3* by PEG-mediated transformation. Fluorescence signals from GFP, mCherry-OsRac3, and the merged images are shown. Free GFP was used as a control. Bars, 10 μm

To examine the spatial expression of *OsLHT1*, real-time PCR analysis was carried out to analyze the transcript levels of *OsLHT1* in different organs of 14-week-old plants, including root, stem (lower internodes), flag leaf sheath, flag leaf and young panicle. The result showed that *OsLHT1* was expressed in all of the rice organs tested, with the lowest expression observed in the lower internodes (Fig. 4a). Further, a more detailed expression of *OsLHT1* at different developmental stages was obtained from the electronic Fluorescent Pictograph (eFP) Browser [25], indicating that *OsLHT1* exhibited high expression in the developing inflorescence and seed (Additional file 1: Figure S4).

To examine the subcellular localization of OsLHT1, the OsLHT1-GFP fusion proteins under the control of the 35S promoter was constructed and transiently expressed in rice protoplasts. After 16–20 h culture, the GFP (green fluorescent protein) fluorescence signal was observed. Protoplasts expressing GFP alone typically showed green fluorescence throughout the cytosol (Fig. 4b). In contrast, in cells expressing OsLHT1-GFP, the green fluorescence was confined to the plasma membrane, co-localized with the plasma membrane-localized small GTPase OsRac3 (mCherry-OsRac3) (Fig. 4b) [26]. The result suggested that OsLHT1 was targeted to the plasma membrane.

### Phylogenetic analysis of *OsLHT1*

**Fig. 5:**
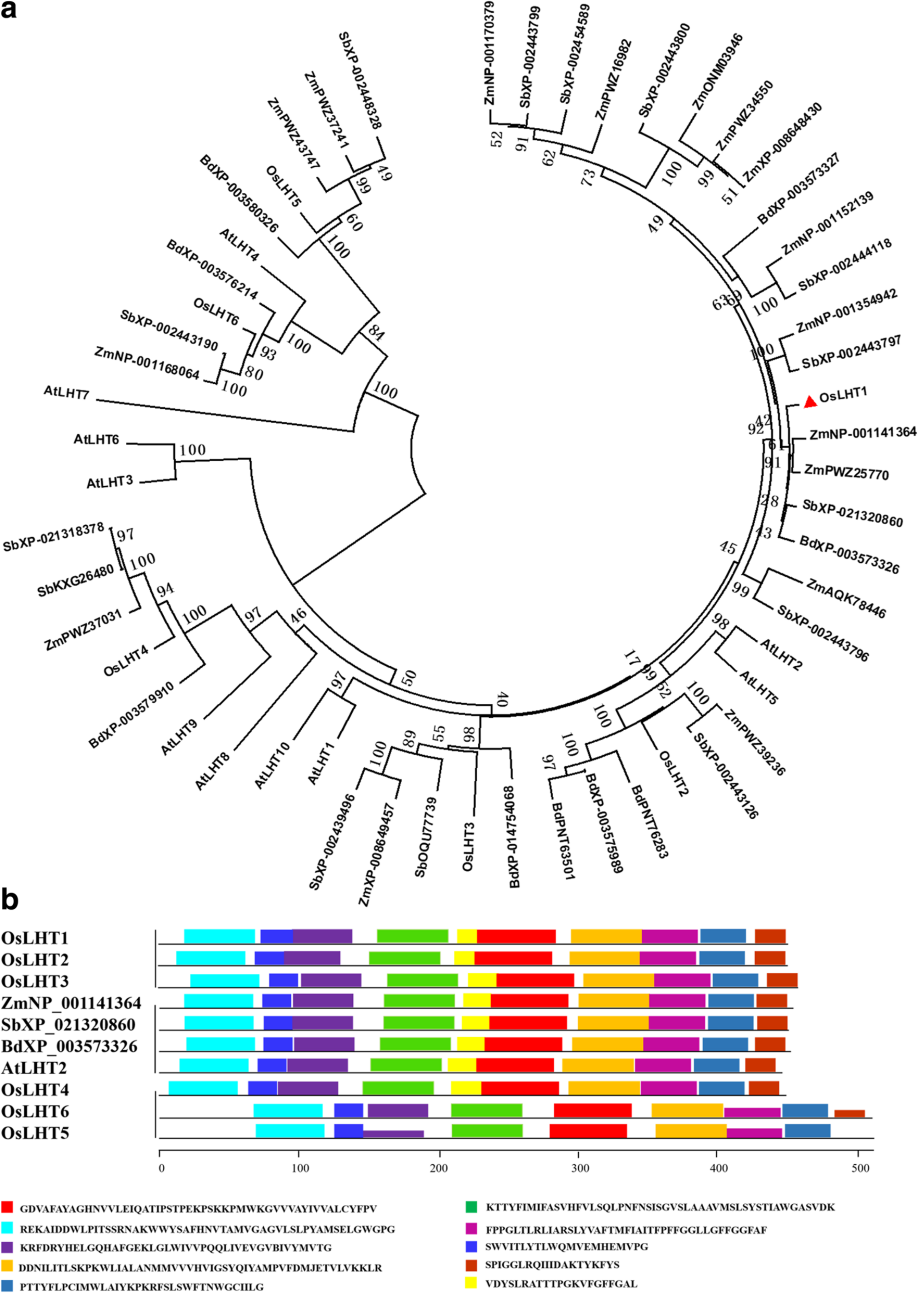
Phylogenetic relationship and protein motifs of OsLHT1 and its homologs. **a** Phylogenetic tree of OsLHT1 and its homologs from maize, sorghum, *Brachypodium* and *Arabidopsis*. With the exception of OsLHT1–6 and AtLHT1–10, the first two letters of each protein label represent the abbreviated species name, followed by GenBank accession number. Zm, *Zea mays*; Sb, *Sorghum bicolor*; Bd, *Brachypodium distachyon*. The phylogenetic tree was constructed by Mega 6.0 software using ClustalW for the alignment and the neighbour-joining method for the construction of the phylogeny [28]. The bootstrap values, shown at the nodes, are percentages for 1000 replications. The red triangle marks the OsLHT1. **b** Schematic representation of conserved motifs in OsLHT1–6 and its closest homologs in maize, sorghum, *Arabidopsis* and *Brachypodium*. Each motif is represented by different color boxes. The order of the motifs corresponds to their position within individual protein sequences

OsLHT1 is a member of LHT subfamily that belonged to the amino acid transporter (ATF) family. 6 and 10 *LHT* genes were identified in rice and *Arabidopsis* genome, respectively [14, 27]. We further used the OsLHT1 sequence as a query to search for its homologs in other monocots, and 16, 14 and 9 homologs were obtained from maize, sorghum and *Brachypodium distachyon*, respectively. These proteins, together with LHTs in rice and *Arabidopsis*, were used for phylogenetic analyses. The resulting phylogenetic tree was mainly divided into two clades, in which OsLHT1 to OsLHT4 belong to one clade, OsLHT5 and OsLHT6 constitute another clade (Fig. 5a). Among the six OsLHTs, OsLHT1 has highest homology to OsLHT2 with identity 76%. The identity of OsLHT1 with its closest homolog in maize (NP_001141364, 90%), sorghum (XP_021320860, 92%) and *Brachypodium distachyon* (XP_003573326, 91%) are much higher with that in *Arabidopsis* (AtLHT2, 78%). Furthermore, the conserved motifs of the six OsLHTs and the closest homologs of OsLHT1 were predicted using the MEME motif search tool. With the exception of OsLHT5 and OsLHT6, the other LHTs have same motifs (Fig. 5b).

## Discussion

AtLHT1 is the first member identified in the LHT subfamily belonging to the amino acid transporter (ATF) family [18]. Uptake measurements in yeast suggested that AtLHT1 transported Lys and His most efficiently among the 13 different amino acids tested, and the *K*_m_ values for Lys (175 μM) and His (400 μM) were much lower than that for the third most-active substrate, Leu (11 mM), so it was regarded as a Lys and His selective transporter [18]. However, studies on the transport properties of other members of LHT family suggested that LHTs were able to transport a wide spectrum of amino acids, including neutral, basic and acidic amino acids, and exhibited preferential selectivity for neutral and acidic amino acids with high affinity [20, 21, 29]. For example, uptake analysis in yeast revealed that AtLHT2 transported aspartate and proline with high affinity (*K*_m_ = 72, 13 μM, respectively), and competition experiments showed that other neutral and acidic amino acids, but not basic amino acids, act as efficient competitors for proline and aspartate uptake [20]. Furthermore, Perchlik et al. [21] studied AtLHT6 function *in planta* and found that knockout of *AtLHT6* significantly reduced the uptake of acidic and some neutral amino acids, but not basic amino acids. There are 6 LHT members existing in rice [14], but their substrate specificity has not yet been determined until now. OsLHT1 has been reported as a His transporter previously [22], and its substrate specificity was analyzed in *Xenopus* oocytes in the present study. We found that OsLHT1 could mediate the transport of various amino acids with different characteristics (Fig. 1a, b). Different from AtLHT1 expressed in yeast [18], the Lys transport activity of OsLHT1 was very weak relative to His (Fig. 1a, b). Moreover, the neutral amino acid Asn, but not the basic amino acid His, was the best substrate for OsLHT1 among the amino acids tested (Fig. 1a, b), so the substrate profile of OsLHT1 resemble that of AtLHT2 and AtLHT6 [20, 21]. However, it should be noted that OsLHT1 and AtLHT2 differ in substrate specificity and affinity. The *K*_m_ value of OsLHT1 for its best substrate Asn is 583 ± 51 μM (Fig. 1c), which is one order of magnitude higher than the values of AtLHT2 for Pro and Asp (*K*_m_ = 13, 72 μM, respectively) [20]. The difference in *K*_m_ values between OsLHT1 and AtLHT2 may imply their different roles *in planta*, which will require further analysis in future study. Overall, given the substrate specificity of the LHTs identified so far, it could be concluded that the LHTs were not Lys and His selective transporters, but ones with broad substrate specificity and with preference for neutral and acidic amino acids.

The substrate profile of OsLHT1 was determined in *Xenopus* oocytes under pH 5.4 condition. At pH 5.4, basic amino acids such as His are positively charged, while acidic amino acids such as Asp and Glu carry mainly negative net charges. However, all these amino acids with opposite charges could be transported by OsLHT1 (Fig. 1a, b). On the other hand, Lys and Arg carrying like charge as His were not efficiently transported by OsLHT1 (Fig. 1a, b). So it is supposed that net charge is not a major determinant of substrate recognition for OsLHT1. Besides charge, individual amino acids differ with respect to geometry, which might play a crucial role in substrate recognition by OsLHT1.

OsLHT1 was localized at the plasma membrane in rice protoplasts (Fig. 4b), which is consistent with its function as an amino acid transporter, and also agrees with previous work by Liu et al. [22] and Whiteman et al. [30]. In Liu’s study, the *OsLHT1-GFP* fusion construct was expressed transiently in onion epidermal cells, and green fluorescence was detected almost exclusively at the plasma membrane [22]. Whiteman et al. [30] isolated rice plasma membrane and tonoplast proteins, and found that OsLHT1 specific peptides were present in plasma membrane fractions but not in tonoplast.

OsLHT1 was expressed in roots (Fig. 4a, Additional file: Figure S4), and could transport amino acids both in yeast and *Xenopus* oocytes (Fig. 1a) [22], which implied that it might be capable of mediating amino acids acquisition from soil. However, given the facts that: (1) free amino acid concentrations found in the bulk soil solution vary greatly, and rarely exceed 50 μM [31–34]; (2) the *K*_m_ value of OsLHT1 for the most active substrate, Asn, was 583 ± 51 μM (Fig. 1c), which is much higher than the free amino acid concentrations in most soil solution; (3) a large number of microorganisms exist in soil, which are the superior competitors for free amino acids in comparison to plant roots, especially at low soil amino acids concentrations [35–37], we reasoned that the main function of OsLHT1 *in planta* might not be to acquire amino acids from soil, which needs to be verified in future studies. On the other hand, the absorbed inorganic N was converted into amino acids in roots or mature leaves (source), which are the major transport forms of N within plants, and were transported to various metabolically active organs (sink) such as root tips, young leaves, flowers and seeds. The source-to-sink translocation of amino acids is involved in several processes such as phloem loading, import into sinks and xylem-to-phloem transfer, which require the mediation of multiple amino acid transporters [11]. The amino acid permeases (AAPs) have been reported to be involved in the source-to-sink transport of amino acids [38–40]. For example, *AtAAP8* is expressed in the source leaf phloem, and loss-of-function of AtAAP8 reduced a broad spectrum of amino acid phloem loading and partitioning to sinks, which in turn led to decreased silique and seed numbers in *ataap8* plants [40]. OsLHT1 was expressed in various shoot organs such as leaf, leaf blade, and localized to the plasma membrane (Fig. 4a, b), implying its function in amino acids translocation *in planta*. Furthermore, a lack of OsLHT1 function significantly inhibited plants development and reduced seed yields (Figs. 2e, 3a), so we supposed that OsLHT1 functions mainly in amino acids transport within plants, but the process it mediated will need further investigations in future.

## Conclusions

Amino acid transporters mediate root amino acids uptake from soil, and their translocation within plants. Research on plant amino acid transporters had a strong focus on *Arabidopsis*, while our understanding of them is generally scant in rice. OsLHT1 was found to serve as a histidine transporter in yeast, but its substrate profile and function *in planta* are unclear. In the present study, we analyzed the substrate selectivity of OsLHT1 in *Xenopus* oocytes, and found that OsLHT1 could transport a broad spectrum of amino acids with preference for neutral and acidic amino acids. Spatial expression and subcellular analysis showed that *OsLHT1* was expressed in various rice organs, and was targeted to the plasma membrane. A loss-of-function of OsLHT1 inhibited rice root and shoot growth, and markedly reduced grain yields. This study will further our understanding of the substrate specificity of OsLHT1 and its roles in rice growth promotion and yield formation.

## Methods

### Plant materials

The wild-type rice(cv. Nipponbare) and two *oslht1* mutants were used in this study. The wild-type rice was ordered from rice resources conservation center of Guangxi University. The *oslht1* mutants were constructed using the CRISPR-Cas9 gene-editing technology in our lab (see below), and its cultivation complies with China’s legislation on genetically modified plants.

### Generation of *OsLHT1* knockout lines

The web-based tool CRISPR-P 2.0 software (http://crispr.hzau.edu.cn/CRISPR2/) was used to design target sites of *OsLHT1*. Two target sequences were 5′-TAACTACCCACCGGCCAAGG-3′ and 5′-TTCCCATCACGTCGTCCAGG-3′, respectively. The pCRISPR-*OsLHT1* plasmid was constructed as described by Ma et al. [41]. Briefly, two sgRNA expression cassettes containing target sequence were constructed by overlapping PCR, and were cloned into pYLCRISPR/Cas9Pubi with *Bsa* I restriction enzyme, resulting in the pCRISPR-*OsLHT1.*The resulting constructs were introduced into Nipponbare via *Agrobacterium tumefaciens*-mediated transformation. The T0 and T1 homozygous mutants were screened by PCR using primer pairs flanking two *OsLHT1*-specific target sites. Two independent knockout lines of *OsLHT1* were selected for further phenotypic analysis.

### Morphological characterization

Seedlings of wild-type and *oslht1* plants were exposed to 0.5 mM CaCl_2_ solution at 28 °C for 5 d (12-h-light and 12-h-dark condition), root length was measured by a ruler at different days. Field tests of rice were carried out at the experimental paddy field of Guangxi University in 2018, germinated seeds of wild type and mutants were cultured in the field. After three-leaf period, plants were transplanted to experiment field in March and harvested at the end of July. Agronomic traits including the plant height, stem length, tiller number, the grain yield per plant, panicle number per plant and grain number per panicle were recorded. The brown rice was weighed, and the 1000-grain weight and grain yield per plant were calculated.

### RNA isolation and gene expression analysis

To investigate the expression pattern of *OsLHT1*, total RNA was extracted from different organs of 14-week-old WT rice using Trizol regent (Invitrogen). Reverse transcription was performed using 1μg of total RNA and the first-strand cDNA was reverse transcribed using superscript reverse transcriptase (Invitrogen) according to the manufacturer’s instructions. The primers 5′-TCTTCGGTGGATTCGCCTTC-3′ and 5′-ATGATGCAGATCCAGTTGGTG-3′ for *OsLHT1*, 5′-GGTCAACTTGTTGATTCCCCTCT-3′ and 5′-AACCGCAAAATCCAAAGAACG-3′ for *Histone* were used for qRT-PCR, and conditions for quantitative analysis were as follows: 94 °C for 2 min, 35 cycles of 94 °C for 15 s, 60 °C for 20s and 72 °C for 30s and final extension at 72°Cfor 10 min. The gene transcript levels in each sample were normalized to that of the housekeeping gene *Histone*. Each experiment was conducted with three biological replicates.

### Subcellular localization of OsLHT1

To investigate the subcellular location of OsLHT1, the full-length coding sequence of *OsLHT1* (LOC_Os08g03350) was amplified with primer pairs: 5′-CCCAAGCTTCGATGGGGACTCAGGTGGCAGATAA-3′ and 5′-CGCGGATCCCTACGAGTAGAACTTGTATGTCT-3′, and was cloned into pYL322-*GFP* vector with *Hind*III and *BamH* I restriction sites for producing *GFP*-*OsLHT1* construct. The plasmid *GFP-OsLHT1* or *GFP* alone (control), along with mCherry-OsRac3 used as a plasma membrane marker [26], were cotransformed into the rice protoplasts by PEG-mediated transformation as previously described [26]. After transformation for 12 to 16 h, the fluorescence signal images were observed via a confocal laser scanning microscope (Tcs sp8; Leica).

### Conserved motif and phylogenetic analysis

Using the OsLHT1 sequence as the query, its homologs in maize, sorghum and *Brachypodium distachyon* were obtained from NCBI database by BLAST program. Multiple sequence alignments of OsLHT1 and its homologs were generated using ClustalW version1.83 with default settings, and the neighbor-joining (NJ) tree was constructed using neighbor-joining (NJ) method of MEGA 6.0 with 1000 bootstrap replications. To better understand the conserved structural features of LHT family proteins, the online tool MEME (Multiple Expectation Maximization for Motif Elicitation) was used to identify the conserved motifs in the encoded LHT family and each motif width was constrained between 3 and 50 residues.

### Electrophysiology

The full-length coding sequence of *OsLHT1* (LOC_Os08g03350) was amplified by high-fidelity PCR from Nipponbare cDNA using gene specific primers 5′-CGCTCAACTTTGGCAGATCTATGGGGACTCAGGTGGCAGATAAC-3′ (initiation codon underlined) and 5′-AGATCCTAGTCAGTCACTAGTCTACGAGTAGAACTTGTATGTC-3′ (termination codon underlined). The resultant PCR product and the *Bgl*II/ *Spe* I linearized vector pT7Ts were recombined with a seamless assembly cloning kit (Vazyme) following the manufacturer’s instructions. The ligated products were then introduced into *E. coli* DH5α and verified by sequencing. The recombinant plasmid DNA was linearized with *Xba* I, and the cRNA was transcribed in vitro using the mMessage mMachine T7 kit (Ambion). *Xenopus* oocytes were prepared as described previously [42]. Oocytes were injected with 23.0 ng of *OsLHT1* cRNA or an equivalent volume of nuclease-free water, using Nanoliter 2000 microinjector, and then incubated for 2–4 days at 19 °C in ND96 solution containing 96 mm NaCl, 2 mm KCl, 1.8 mm CaCl_2_, 1 mm MgCl_2_, 5 mM HEPES (pH 7.4) and 50 mg/L gentamicin. Whole-cell currents were recorded using the TEVC technique (AxoClamp 900A, Molecular devices), and data acquisition, data analysis were carried out using software Clampex, clampfit 10.3 (Molecular devices) and SigmaPlot 12.0 (Systat Software). For current recording as in Fig. 1a and b, oocytes were voltage-clamped at − 70 mV. For current recording as in Fig. 1c, the holding potential was − 40 mV, and Asn-induced currents were measured at membrane potentials from − 140 to + 20 mV in 20 mV increments for 500 ms. The basal superfusing solution contained: 90 mM NaCl, 2 mM CaCl_2_, 2 mM MgCl_2_, and 4 mM MES, and amino acids were added at desired concentrations. The pH of all solutions was adjusted to 5.4 using Tris/MES.

### Statistical analysis

All treatments were repeated at least two times, and the statistical analysis difference was indicated by asterisks. Significant levels was defined ^*^P < 0.05, ^**^P < 0.01, ***P < 0.001.

## Additional file

Additional file 1: **Figure S1.** Transcript level of *OsLHT1* in the wild-type and *oslht1* plants. **Figure S2.** Germination rates of the wild-type and *oslht1* seeds. **Figure S3.** Grains of the wild-type and *oslht1* mutants. **Figure S4.** Transcript level of *OsLHT1* in the developing organs. **Table S1.** Raw qRT-PCR data in this study (Fig. 4a). (DOCX 586 kb)

## Data Availability

All data and materials generated or analyzed during this study are included in this article or are available from the corresponding author on reasonable request.

## Abbreviations

BLAST: Basic Local Alignment Search Tool
GFP: Green Fluorescent Protein
LHT: Lysine and Histidine Transporter
PCR: Polymerase Chain Reaction
TEVC: Two-Electrode Voltage Clamp
