# Supplementary material for: Disruption of an amino acid transporter LHT1 leads to growth inhibition and low yields in rice
Source: BMC Plant Biol. 2019 Jun 20;19:268. doi: 10.1186/s12870-019-1885-9 (PMC6584995; doi:10.1186/s12870-019-1885-9)
Supplement: Supplementary file 1 — Figure S1. Transcript level of OsLHT1 in the wild-type and oslht1 plants. Figure S2. Germination rates of the wild-type and oslht1 seeds. Figure S3. Grains of the wild-type and oslht1 mutants. Figure S4. Transcript level of OsLHT1 in the developing organs. Table S1. Raw qRT-PCR data in this study (Fig. 4a). (DOCX 586 kb) [file 12870_2019_1885_MOESM1_ESM.docx]

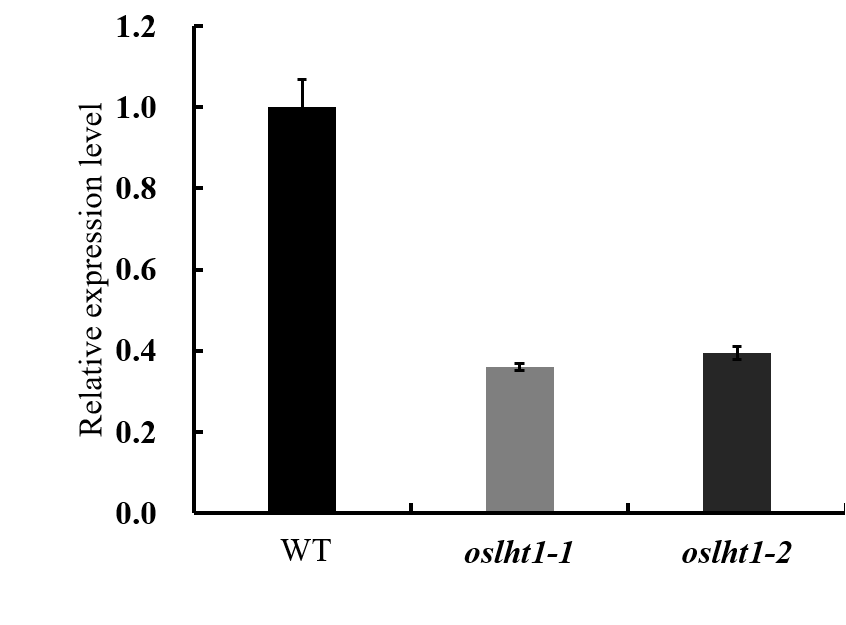


**Figure S1** Transcript level of *OsLHT1* in root of the wild-type and *oslht1* plants, analyzed by qRT-PCR. The data are shown as the mean ± SD (n = 3).


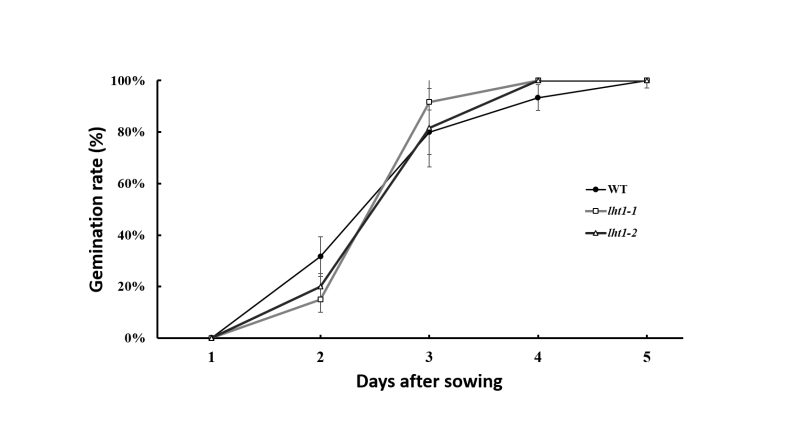


**Figure S2** Germination rates of the wild-type and *oslht1* seeds.

Seeds of wild-type and *oslht1* plants were cultured in 0.5mM CaCl_2_ solution at 28°C for 5 days. Results are averages of 3 independent experiments. Error bars indicate standard deviations.


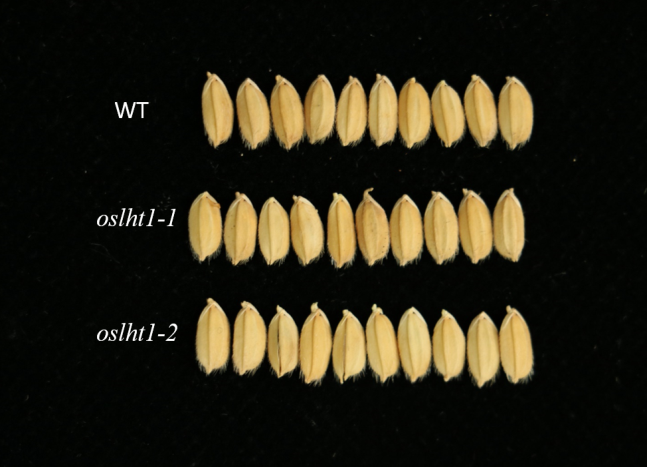


**Figure S3** Grains of the wild-type and *lht1* mutants.


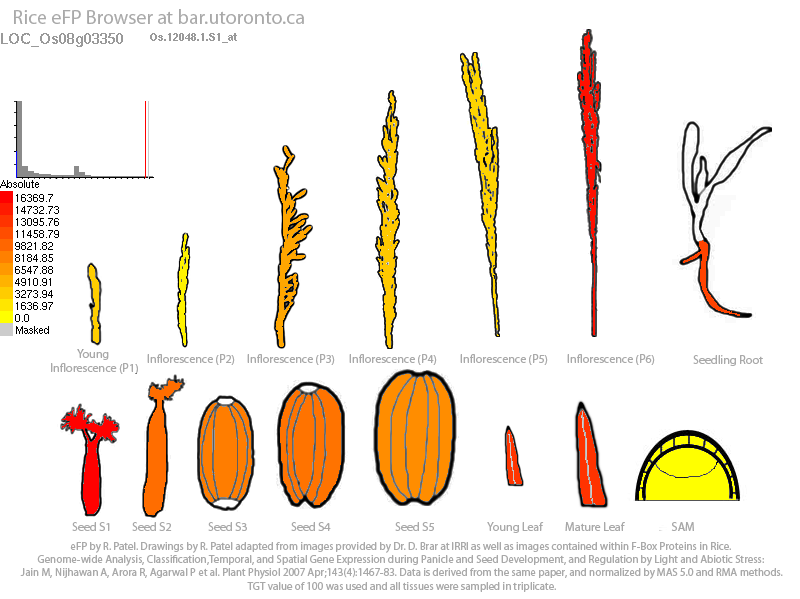


**Figure S4** Transcript level of *OsLHT1* in the developing organs. Pictograms were extracted from the eFP browser database [25].

Table S1 Raw qRT-PCR data in this study (Fig. 4a).

|  | Ct value of *Histone* | Ct value of *OsLHT1* |
| --- | --- | --- |
| **Young panicle** | **18.14** | **19.65** |
|  | **18.66** | **21.93** |
|  | **18.17** | **21** |
|  | **18.95** | **20.89** |
| **Flag leaf** | **18.41** | **19.5** |
|  | **18.14** | **19.49** |
|  | **18.53** | **19.95** |
|  | **18.21** | **19.57** |
| **Flag leaf sheath** | **18.48** | **20.28** |
|  | **19.03** | **21.31** |
|  | **18.12** | **19.95** |
|  | **18.4** | **21.2** |
| **Lower internode** | **19.03** | **24.55** |
|  | **19.43** | **26.74** |
|  | **19.01** | **24.38** |
|  | **19.24** | **24.05** |
| **Root** | **19.34** | **21.17** |
|  | **19.15** | **21.38** |
|  | **18.83** | **21.2** |
|  | **19.61** | **21.41** |
